# Saliva as a sampling source for the detection of leukemic fusion transcripts

**DOI:** 10.1186/s12967-014-0321-z

**Published:** 2014-11-19

**Authors:** Dongmei Chen, Najie Song, Runfang Ni, Jiangning Zhao, Jiasheng Hu, Quanyi Lu, Qingge Li

**Affiliations:** State Key Laboratory of Cellular Stress Biology, State Key Laboratory of Molecular Vaccinology and Molecular Diagnostics, Engineering Research Centre of Molecular Diagnostics, Ministry of Education, School of Life Sciences, Xiamen University, Xiamen, Fujian 361102 China; Shenzhen Research Institute of Xiamen University, Shenzhen, Guangdong 518057 China; Zhongshan Hospital of Xiamen, Xiamen University, Xiamen, Fujian 361004 China

**Keywords:** Leukemia, Leukemic fusion transcript, Saliva, Molecular diagnosis, Minimal residual disease

## Abstract

**Background:**

Saliva has long been used as a sampling source for clinical diagnosis of oral disease such as oral squamous cell carcinoma, or therapeutic drug monitoring. The aims of this study was to ascertain if saliva RNA could be stored at room temperature and to study if saliva could be a convenient source for fusion transcripts in leukemic patients.

**Methods:**

This is a cross-sectional diagnostic study. We first developed a Saliva RNA tube for stable storage of whole saliva RNA at room temperature. Then we detected the leukemic fusions in the whole saliva from seven leukemic patients and twenty healthy volunteers, and compared with the results obtained from the bone marrow of the patients.

**Results:**

Human gene transcripts could be reproducibly detected in the whole saliva for at least four weeks when stored in the developed composition at room temperature. Concordant results of the fusion transcripts were obtained between the saliva and the bone marrow in the seven leukemic patients and no fusions were detected in the healthy controls.

**Conclusions:**

The results support our hypothesis that human whole saliva could be a reliable and convenient sampling source for the detection of leukemic fusions.

## Background

Leukemic fusion genes generated by chromosome rearrangement are hallmarks of leukemia [1,2]. For examples, the chromosomal abnormalities, t(9;22)(q34;q11) (BCR-ABL), t(15;17)(q22;q12) (PML-RARα) and t(8;21)(q22;q22) (AML-ETO), are present in 90% to 95% of chronic myeloid leukemia (CML) cases, in more than 99% of acute promyelocytic leukemia (APL) cases and in 8% to 20% of acute myeloid leukemia (AML) cases, respectively. Detection of fusion transcripts is important in determining the disease status, prognosis, and tailored therapy [3,4]. Leukemic fusion transcripts are routinely detected by using whole blood or bone marrow as the sampling sources. However, either whole blood or bone marrow is difficult to collect, store and transport, and it is often the case that highly trained personnel and expensive facility are required for sampling it. Moreover, the invasive nature of the sampling procedure renders repeated sampling a challenging task, especially, in the case of close monitoring of disease status.

Saliva has long been used as a sampling source for clinical diagnosis [5-13]. Saliva offers advantages over blood and bone marrow for its non-invasive nature, easy storage and transportation, cost-effectiveness, and safe handling [7]. Previous studies showed that 74% of the total DNA in saliva comes from human leucocytes of various types [14,15]. We thus envisioned that the leucocytes in saliva might be a convenient source for the fusion transcripts in leukemic patients. In this cross-sectional diagnostic study, we aims to ascertain if saliva RNA could be stored stably at room temperature and to study if saliva could be a reliable source for the detection of fusion transcripts in leukemic patients.

## Material and methods

### Subjects

We recruited 20 healthy individuals from our laboratory and 7 leukemic patients from Zhongshan Hospital of Xiamen for saliva collection in this study between September 2013 and April 2014. The study protocol for sample collection was approved by Research Ethics Committee of Xiamen University and an informed consent was signed for each patient. All subjects were confirmed by licensed dentists to have no mucosal lesion or inflammation in the oral cavity. The oral mucosa appeared healthy, without erythema and epithelial desquamations. The patients were pathologically diagnosed by certified pathologists as CML (n = 2), APL (n = 4) and AML (n = 1), respectively. These patients were examined for BCR-ABL, PML-RARα or AML-ETO transcripts using bone marrow samples in the hospital. Also, the BCR-ABL was typed into p190 and p210, and PML-RARα was typed into S, L and V using the same samples. All these molecular assays using bone marrow samples were performed routinely in the hospital through a commercial service. Thirteen of the 27 samples were male and 14 were female. The median age of the subjects was 25 years old (range from 21 to 51 years old). Both of the patients and healthy individuals were the Han nationality.

### Sample collection

To collect human whole saliva for easy transportation, we developed a Saliva RNA tube for stable storage of salivary RNA at room temperature based on a commercial kit [16]. The formed Saliva RNA tube contained 2 mL of formulated reagent (stabilizer) for RNA stabilization. The stabilizer comprises 4% sodium dodecyl sulfate (SDS) and 50 mM sodium citrate at pH 6.8. The principle of this method is that SDS, an anionic detergent, is used to inhibit ribonuclease activity in a weakly acid buffer. Before saliva collection, all subjects were asked to rinse their mouths with water and 2 mL of whole saliva were collected into the Saliva RNA tube after 30 minutes. Then, the saliva was mixed with the stabilizing liquid from the cap by inversing the tube upside down three times.

### RNA extraction and reverse transcription

From the Saliva RNA tube, 500 μL of saliva solution was transferred to a 1.5-mL micro-centrifuge tube, to which 8 μL of proteinase K was added. After a thorough mixing step by inversion, the mixture was incubated overnight at 50°C in a water bath followed by heating at 90°C for 15 min, then cooled down to room temperature. To the above mixture, 21 μL of KCl (2.5 M) was added and mixed thoroughly by inversion. The mixture was kept on ice for 10 min and centrifuged (13,000 × g) at 4°C for 10 min. The supernatant was carefully transferred to a fresh micro-centrifuge tube, to which two volumes of cold 95% ethanol was added and mixed thoroughly by inversion. After storage at −20°C for 1 h, the mixture was centrifuged (13,000 × g) at 4°C for 15 min. The pellet was dissolved into 350 μL of buffer RL (RNAprep Pure Micro Kit, Tiangen, Beijing, China), to which 350 μL of cold 70% ethanol was added and mixed. RNA purification was performed immediately using Tiangen RNAprep PureMicro kit. Reverse transcription was carried out using the PrimeScript® RT reagent Kit (TaKaRa, Dalian, China).

### Quantitative real-time reverse-transcription (qRT)-PCR

The transcripts of a commonly used reference gene *β*-*actin* were detected using a qRT-PCR assay. The 25-μL reaction consisted of 67 mM Tris–HCl (pH7.8), 6.7 μM EDTA, 16.6 mM (NH_4_)_2_SO_4_, 0.085 mg/mL bovine serum albumin, 2 mM MgCl_2_, 25 μM of each dNTP, 400 nM of each primer, 100 nM of each probe, 1 U of Taq DNA polymerase and 5 μL of cDNA template. qRT-PCR was carried out on the Stratagene Mx3005*P* Real-Time PCR System (Agilent Technologies, Santa Clara, CA) under the following thermocycling conditions: 95°C for 10 min, followed by 50 cycles of 95°C for 15 s, 58°C for 20 s, and 72°C for 30 s. Each sample was performed in duplicate (sic passim). Leukemic fusion transcripts were detected using Q-Fusion (QuanDx, San Francisco, CA), a multiplex qRT-PCR assay that detects 30 common leukemic fusions. BCR-ABL fusion was then typed using a BCR-ABL typing (p190, p210) assay, and PML-RARα was typed using a PML-RARα typing (L, V, S) assay (QuanDx), respectively. qRT-PCR was performed in the Stratagene Mx3005*P* Real-Time PCR System following the manufacturer’s protocol.

## Results

### Stability of RNA extracted from the whole saliva stored at room temperature

**Figure 1:**
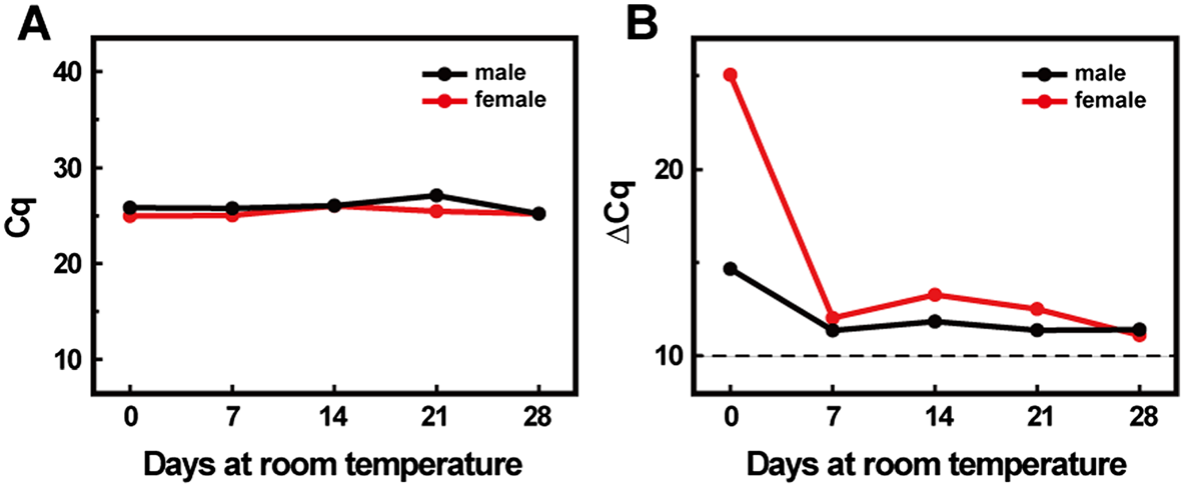
**The stability of RNA extracted from the whole saliva stored at room temperature.** Saliva was collected respectively from two healthy individuals and stored in the Saliva RNA tubes for up to four weeks. Total RNA was extracted at different time intervals. The transcripts of *β*-*actin* were detected using the qRT-PCR assay at different weeks. **(A)** The Cq values of the the two samples at different weeks. The Cq for the male and female saliva were calculated to be 26.00 ± 0.70 and 25.34 ± 0.43, respectively. **(B)** The ΔCq values of the two samples at different weeks. ΔCq = Cq_RT(−)_ – Cq_RT(+)_, where Cq_RT(+)_ was the Cq values obtained from the extracted RNA with reverse transcription, whereas Cq_RT(−)_ was the Cq values obtained without reverse transcription.

We first tested whether human gene transcripts in the whole saliva could be reproducibly detected after storage at room temperature. For this purpose, saliva samples were collected from two healthy individuals, one male and one female. RNA was extracted and subjected to qRT-PCR for *β*-*actin* mRNA analysis at intervals between 0 to 4 weeks. Our results showed that the quantification cycle (Cq) values of *β*-*actin* mRNA kept nearly constant for 4 weeks (Figure 1A), demonstrating that the mRNA level of *β*-*actin* in the saliva remained largely unchanged during the 4-week storage at room temperature. The differences of Cq (ΔCq) between the RT(+) and RT(−) were larger than 10 (Figure 1B), indicating that there was negligible (<0.1%) contamination of human genomic DNA [17]. We thus concluded that human gene transcripts could be detected in the whole saliva and remain stable for at least four weeks when stored in the Saliva RNA tube at room temperature.

### Detection of fusion transcripts in saliva from both healthy individuals and leukemia patients

**Figure 2:**
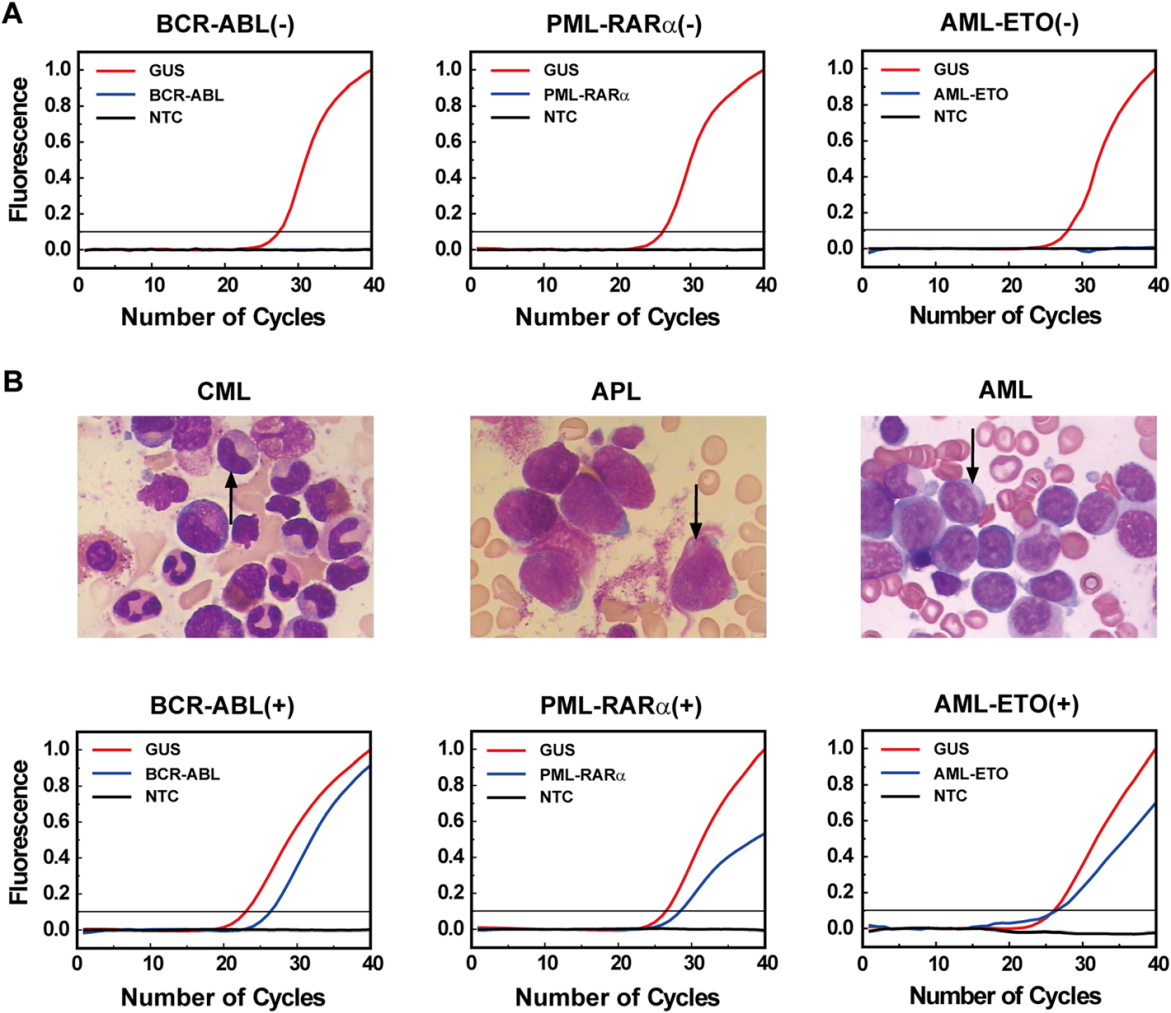
**Detection of fusion transcripts in saliva from both healthy individuals and leukemic patients. (A)** qRT-PCR results of the representative healthy individuals for the detection of BCR-ABL, PML-RARα, and AML-ETO. **(B)** qRT-PCR results of the three types of leukemia patients for the detection of BCR-ABL, PML-RARα, and AML-ETO. The upper panel showed the pathological results for the patients and the lower panel showed the corresponding qRT-PCR results. In all detections, *GUS* was used as the reference gene and water was used as the no-template control (NTC).

We then tested whether leukemia-specific fusion gene transcripts could be detected in the whole saliva of the leukemic patients. To this end, whole saliva was collected from each of the 27 recruited individuals including 20 healthy individuals and 7 leukemic patients. The saliva samples obtained were renumbered. After RNA extraction, the samples were subjected to the Q-Fusion assay in a blind format. The results showed that 20 samples were negative for leukemic fusions, 2 positive for BCR-ABL, 4 positive for PML-RARα, and 1 positive for AML-ETO (Figure 2). By referring to the results from the hospital, a complete agreement between the two assays was achieved. The qRT-PCR results also agreed with the pathological diagnosis, i.e., the 2 samples positive for BCR-ABL were from the CML patients, 4 positive for PML-RARα were from the APL patients, and 1 positive for AML-ETO was from the AML patient (Figure 2). These results demonstrated that leukemic fusion transcripts could be accurately detected in the whole saliva of the leukemic patients.

### Determination of the type of fusion transcripts in saliva from leukemic patients

**Figure 3:**
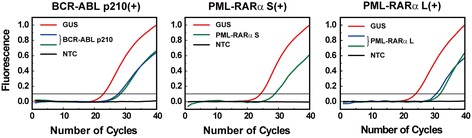
**Determination of the type of fusion transcripts in saliva from leukemic patients.** qRT-PCR results of the CML and APL patients for the typing of BCR-ABL and PML-RARα, respectively. In all detections, *GUS* was used as the reference gene and water was used as the no-template control (NTC).

Using the same saliva samples, we further determined the type of the two BCR-ABL samples from the CML patients and the four PML-RARα samples from the APL patients. The results showed that both the CML patients had p210 subtype for BCR-ABL, three APL patients had S subtype, and one APL patient had L subtype for PML-RARα (Figure 3). In comparison with the results obtained from the hospital where the typing assays using bone marrow samples were performed routinely through a commercial service, a complete concordance was again achieved. We thus concluded that the type of the fusion transcripts could also be correctly determined in the whole saliva of the leukemic patients. Taken together, we confirmed that leukemic fusion transcripts could be detected in the saliva of the leukemic patients.

## Discussion

This is the first report of using saliva as a sampling source for the detection of leukemic fusion transcripts. It has long been regarded that salivary RNA is from oral epithelial cells and oral micro-organisms [18]. Therefore salivary RNA has been already used for diagnosis of oral carcinoma and oral infection. Recent studies on the use of salivary transcriptomic biomarkers for detection of pancreatic cancer [19] and breast cancer [12] suggest that salivary RNA might also be used to detect those conditions other than oral diseases.

In this preliminary study, we demonstrated that leukemic transcripts could be reliably detected from salivary RNA and the detection results from saliva fully agreed with those obtained from bone marrow. The small sampling of this study precludes us to calculate the sensitivity or specificity values of the testing, however, the facts that human RNA in saliva could be stably stored and repeatedly detected support the diagnostic value of our study.

Saliva collection is a truly non-invasive sampling format, and repeated sampling can be easily performed. Because no risk of infection exists and no expertise is needed, sampling can be carried out at home by the patient, stored at room temperature, and sent to the hospital through surface mail. Not only the cost is substantially reduced, the way of disease management can also be significantly improved.

The success of the detection of fusion transcripts using saliva as sampling source by qRT-PCR might also be extended to other diagnostic approaches for leukemia diagnosis such as cytogenetic analysis and fluorescence *in situ* hybridization. Further studies are now being undertaken to clarify the association of fusion transcript level among saliva, blood, and bone marrow in a quantitative format. Such an association might allow the potential use of saliva for the detection of the minimal residual disease in leukemia.

## Conclusions

This study developed a composition for stable storage of saliva RNA at room temperature and confirmed that saliva can be used as a sampling source for the detection of leukemic fusion transcripts. Saliva offers advantages over blood and bone marrow for its non-invasive nature, easy storage and transportation, cost-effectiveness, and safe handling. We thus expect that the adoption of saliva sampling would advance the development of new paradigms for diagnosis, prognosis, and tailed therapy of leukemia.
